# A versatile *ex vivo* technique for assaying tumor angiogenesis and microglia in the brain

**DOI:** 10.18632/oncotarget.6550

**Published:** 2015-12-11

**Authors:** Ali Ghoochani, Eduard Yakubov, Tina Sehm, Zheng Fan, Stefan Hock, Michael Buchfelder, Ilker Y. Eyüpoglu, Nicolai Savaskan

**Affiliations:** ^1^ Laboratory for Translational Neurooncology, Department of Neurosurgery, Universitätsklinikum Erlangen, Friedrich Alexander University of Erlangen-Nürnberg (FAU), 91054 Erlangen, Germany

**Keywords:** glioblastoma, angiogenesis, neuronal cell death, slice culture, ex vivo

## Abstract

Primary brain tumors are hallmarked for their destructive activity on the microenvironment and vasculature. However, solely few experimental techniques exist to access the tumor microenvironment under anatomical intact conditions with remaining cellular and extracellular composition. Here, we detail an *ex vivo* vascular glioma impact method (VOGIM) to investigate the influence of gliomas and chemotherapeutics on the tumor microenvironment and angiogenesis under conditions that closely resemble the *in vivo* situation. We generated organotypic brain slice cultures from rats and transgenic mice and implanted glioma cells expressing fluorescent reporter proteins. In the VOGIM, tumor-induced vessels presented the whole range of vascular pathologies and tumor zones as found in human primary brain tumor specimens. In contrast, non-transformed cells such as primary astrocytes do not alter the vessel architecture. Vascular characteristics with vessel branching, junctions and vessel length are quantitatively assessable as well as the peritumoral zone. In particular, the VOGIM resembles the brain tumor microenvironment with alterations of neurons, microglia and cell survival. Hence, this method allows live cell monitoring of virtually any fluorescence-reporter expressing cell. We further analyzed the vasculature and microglia under the influence of tumor cells and chemotherapeutics such as Temozolamide (Temodal/Temcad^®^). Noteworthy, temozolomide normalized vasculare junctions and branches as well as microglial distribution in tumor-implanted brains. Moreover, VOGIM can be facilitated for implementing the 3Rs in experimentations. In summary, the VOGIM represents a versatile and robust technique which allows the assessment of the brain tumor microenvironment with parameters such as angiogenesis, neuronal cell death and microglial activity at the morphological and quantitative level.

## INTRODUCTION

Brain vasculature and the angiogenic process play both key roles during development and function of the brain [1] [2]. Apart from physiological processes, aberrant vasculature is involved in certain cerebral pathologies such as stroke, inflammation, multiple sclerosis [3], epilepsy [4], Alzheimer's disease [5] and brain tumors [6, 7]. Angiogenesis is mainly studied in *in vivo*-experiments [8–10]. *In vitro* bioassays are characterized by their reduced or deprived environmental impact and their versatile assessability [2]. Only a few *ex vivo* assays exist to study angiogenesis in an organotypic environment with remaining cellular complexity, organotypic microenvironment [11, 12] and intact neurovascular units [13–19]. There are intrinsic advantages in evaluating the angiogenic process and the vascular morphology in organotypic 3D culture compared to 2D cell cultures. Cell culture assays such as the tube formation, retina, aortic ring and endothelial spheroid assays which use dissociated endothelial cells (mainly human umbilical vein endothelial cells [HUVEC], brain endothelial cells or aortic endothelial cells) or tissue pieces (retina or arterial tissues) are to a certain extent redundant or reflect ectopic angiogenesis independent of the particular conditions present in the brain microenvironment [20, 21]. Such cell culture-based models lost their tissue integrity and are deprived from their physiologic micromilieu, connectivity-dependent signals and their organotypic environment which are important determinants in *in vivo* processes.

Here, we describe the establishment of the *Vascular Organotypic Glioma Impact Model* (VOGIM) presenting a robust and reliable tool to investigate physiological and pathological angiogenesis. In principle, any brain tissue from wild type or transgenic mice or rodents can be facilitated for the organotypic brain slice assay. We provide evidence that the *ex vivo* cerebral vasculature and the intact cell structure resemble closely the *in vivo* environment. Various genetic backgrounds can be used for donor tissues including transgenic animals with respective controls. Genetically identical slices can be produced and brought into the culture. Furthermore, slice cultures can be prepared virtually from any region of the brain [22] or even from peripheral organs like liver, kidneys and many others [23]. Moreover, monitoring of defined cells in brain slices can be achieved by acquiring tissue from transgenic animals expressing fluorescent reporter genes under the control of cell-type specific promoters such as CX3CR1 (for microglia), GFAP (for astrocyte-specificity) or tie1 (for endothelial cells) and by ectopic gene expression through ectopic transfection or viral infection [24]. Furthermore, the impact of tumors on neurons and bystanders is assessable in the VOGIM [11, 25]. Thus, we provide here a reliable and versatile method to investigate tumor angiogenesis and the tumor microenvironment in the VOGIM *ex vivo* culture system.

## RESULTS

### The VOGIM procedure and tumor-induced brain damage

For investigations of the brain tumor angiogenesis we first facilitated organotypic brain slice cultures. This *ex vivo* assay has been previously tested in proof-of-principle assays showing its suitability for remaining an organotypic environment with preserved cellular and extracellular complexity and neurovascular units [14, 15].

**Figure 1:**
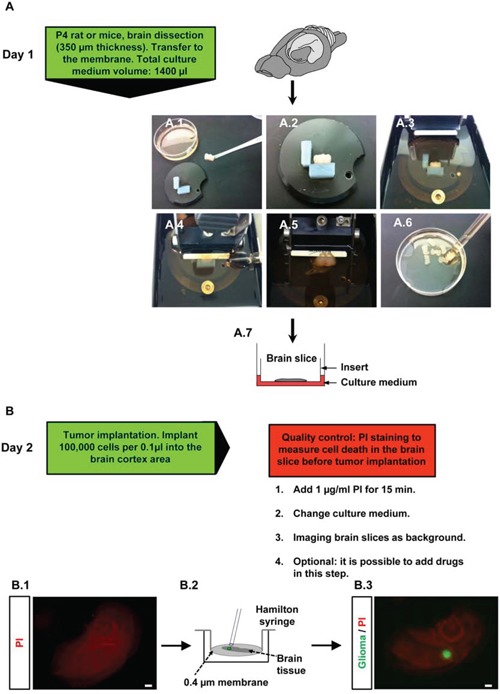
Flowchart and procedure of the VOGIM protocol **A.** Day 1: Isolation of brain tissue and brain dissection (‘slicing’). Green box gives hand-on details of this experimental step. **A.1.** Transfer of trimmed brain tissue to fit onto the specimen disc. **A.2-3.** Fixation of the trimmed brain on the specimen disc, afterwards it is placed into buffer chamber filled with cold preparation medium. **A.4-5.** Sectioning horizontal brain slices in 350 μm thickness. **A.6.** Collection of brain slices with large blunted Pasteur pipette in petri dish filled with cold preparation medium. **A.7.** Transfer of brain slices onto a 0.4 μm pore-size transwell membrane insert which is placed into a six well plate filled with 1400 μl culture medium. **B.** Day 2: tumor implantation step, green and red boxes indicate details and give quality control checkpoints of experimental steps. **B.1.** PI staining and intensity measurement in the brain slices before tumor implantation to evaluate quality. **B.2.** Tumor implantation method is presented. Implantation of 100,000 cells per 0.1 μl into the cortex area with a Hamilton syringe (one μl capacity). **B.3.** Fluorescence microscopic images for the evaluation of invaded tumor cells into brain slice. Scale bar represents 500 μm.

Here, we analyzed the *Vascular Organotypic Glioma Impact Model* (VOGIM) to study different parameters such as tumor growth, tumor cell death, physiological vasculature and tumor-induced angiogenesis. The tissue for the organotypic brain slice assay was derived from 4 days old wild type or transgenic rodents and mice. Brains were cut into 350 μm thick organotypic slices and transferred onto transmembrane inserts for further culturing (Figure 1A). During a resting and stabilization period of one day in culture we analyzed the quality of each section and overall cell survival was determined (Figure 1B). Only those brain sections with intact tissue integrity which showed solely basal cell death were processed further. A critical part of the VOGIM procedure is the tumor implantation. After one day in culture, we started to implant tumor cells into brain sections. For the implantation process we used a micro-syringe which prevents brain tissue damage compared to other application methods (Figure 1B). Approximately 100,000 tumor cells in a volume of 100 nl were implanted into the brain tissue under sterile conditions (Figure 1B, lower column). After tumor implantation, documentation of the developing tumor bulk was performed every second day. One day after the tumor implantation fluorescence microscopy generally shows an undefined, cloud-like tumor. However, several days after implantation the tumor bulk becomes more defined and a clear border is detectable (Figure 1B, lower column).

**Figure 2:**
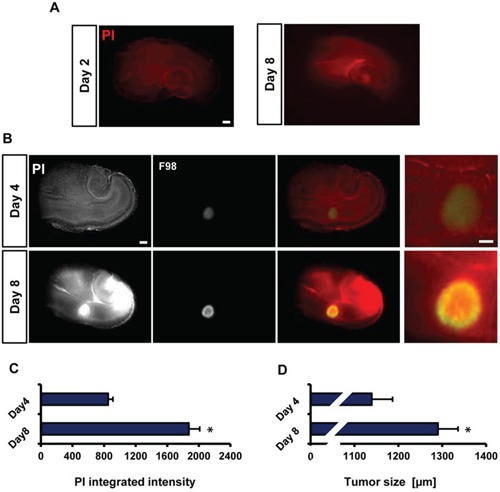
Visualization of tumor growth and cell death in the VOGIM protocol **A.** Representative fluorescence images for PI staining of brain slices to detect cell death after 8 days in culture. Scale bar represents 500 μm. **B.** Representative fluorescence images monitoring tumor growth (green) and cell death (PI) at day 4 and day 8. Scale bar represents 500 μm. **C.** Quantification of cell death (PI intensity quantification) at day 4 and 8 (*n* = 9). **D.** Quantification of tumor size measurements on day 4 and 8 (*n* = 9). Scale bar represents 500 μm. Statistical analysis was performed with Student's *t*-test (*P < 0.05, mean is given ± s.e.m.).

We next tested the general cell viability and survival in brain slices during the VOGIM process after two and eight days in culture. The propidium iodide (PI) staining were utilized as a cell death marker and revealed a low background staining which slightly increased with culture time (Figure 2A). Thus, these results demonstrate that brain tissue is viable and histological intact with the given culture period.

To assess and display tumor growth and tumor-induced cell death in the VOGIM process, we implanted constitutively GFP expressing tumor cells into slices one day after culture (Figure 1B). Tumors size and cell death increased over time and reached highest levels at day 8 in culture. (Figure 2B). PI intensity measurement showed higher intensity levels in brain slices implanted with tumor cells on day 8 in comparison to day 4 (Figure 2B). The PI intensity signal increased three fold on day 8 compared to the levels at day 4 (Figure 2C). This is well in line with previous reports on tumor-induced neurodegeneration *in vivo* [25]. Moreover, tumor size measurements displayed about 150 μm increase in growth on day 8 in comparison to tumor size at day 4 (Figure 2D). In conclusion, the results propose that the VOGIM technique display differences in both tumor cells growth and cell death in the brain slices.

### The VOGIM resembles the vascular architecture of the brain

To establish a reliable quantification technique for the evaluation and measurement of vessel length, branching and diameter, we compared two widely used and established methods. First, we assessed the grid-overlay technique to the VOGIM method. The principle of the grid-overlay technique is based on a virtual grid placed onto the image thereby allowing to measure the number crossing vessels per area. As a matter of fact, the number of vessels correlates with the number of grid crossing points. The crossing points can be counted in two ways: observer-dependent in a manual manner or automatized with software-based applications such as Photoshop or freely available NIH Image J. Moreover, the overall number of vessels can be calculated as well as any area of interest or tumor zones (Supplementry Figure 1A). To analyze vessel-density in cortex or randomized areas, samples were blinded for the counting procedure by coding all images of the different experimental groups (Supplementry Figure 1A). In addition, we considered a second evaluation method based on the free-software NIH Image J (see material and methods for details). The Image J application mainly calculates identified vessel trees generated from immune-positive signals (Supplementry Figure 1B). This approach gives a higher level of data quality and controls for artefacts or certain spots which can be easily identified and excluded for further quantification procedure. The application offers a great number of possibilities and parameters to measure and detect. We applied the following parameters for vessel analysis: *total length, number of junctions* and *number of branches* (Supplementry Figure 1B). The quantity of vessel diameter is a standard approach in vascular investigation. Changes in vessel diameter can also be measured by Image J or the ZEN software (Zeiss, Germany) manually. The vessel diameter measurements were performed at higher magnification over the entire length of single blood vessels (Supplementry Figure 1C).

### Quantitative vessel assessment in different brain areas

**Figure 3:**
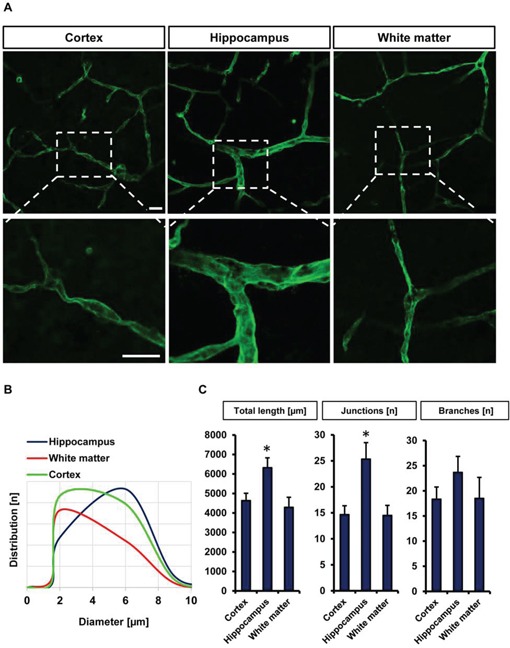
Visualization and quantification of the vasculature in different brain slice areas **A.** Cortex, hippocampus and white matter were candidate to monitor vascularization in different bran slice areas. Laminin antibody was utilized to stain vessels. Scale bar represents 20 μm. **B.** Distribution of vessels diameter (μm) in cortex, hippocampus and white matter (*n* = 6). **C.** Quantification of different vascular parameters total vessels length, number of junctions and branches with Image J Angiogenesis tools plugin. Images with 314 μm × 314 μm size were used for quantification. Statistical analysis was performed with Student's *t*-test (*P < 0.05, error bars represent mean ± s.e.m.).

In the next step we investigated blood vessel parameters in different brain areas of the VOGIM (Figure 3A). For this we immunostained brain slices for laminin. First we evaluated blood vessel diameters in the cortex, hippocampus and in white matter. Interestingly, these analyses revealed region-specific blood vessel diameter distributions (Figure 3A). In the hippocampus, blood vessels diameter ranged between 5 to 7 μm while vessel diameters in the cortex had a range of 2 to 4 μm as well as in the white matter (Figure 3B). Analysis of total blood vessels length displayed significantly higher in the hippocampal area compared to cortex and white matter (Figure 3C). Moreover, the analysis showed significant higher number of junctions in the hippocampus while number of branches displayed slight differences between hippocampus and other areas of the brain slice (Figure 3C). Altogether, our results show that the VOGIM is sensitive enough to display differences of vascular parameters in the various areas of the brain slice as has been found *in vivo* (Heinzer et al., 2008).

### Quantitative assessment of vessels and microglia in peritumoral zones

**Figure 4:**
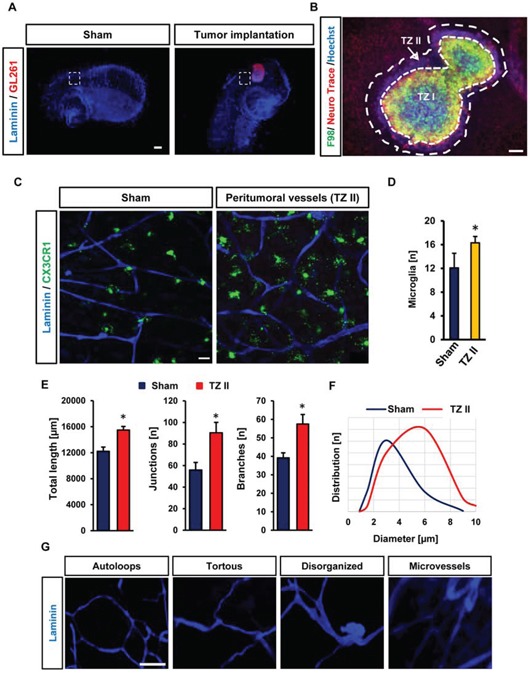
Visualization and quantification of tumor angiogenesis and microglia **A.** CX3CR1-GFP mouse brain slices were utilized to implant RFP expressing murine glioma cells (GL261). For control samples, we implanted only medium at the same position and in the same manner as tumor was implanted. White squares display peritumoral positions or peri-injection area in sham operated slices which were quantified. Scale bar represents 500 μm. **B.** Representative image displaying cell death and tumor zones in the VOGIM. Cell nuclei staining is shown in blue (stained with Hoechst 33258), tumor (green) and neurons (red) are given. Arrow indicates the typical breaking-up of neuronal cell structures in the peritumoral zone (TZ II). The tumor bulk is also termed as Tumor Zone I (TZ I). Scale bar represents 100 μm. **C.** Vascularization in peritumoral area (TZ II). Vessels are stained for Laminin (blue). GFP positive (CX3CR1-GFP mice) cells represent perivascular microglia. Scale bar represents 20 μm. **D.** Number of perivascular microglial cells in TZ II in comparison to sham. Images with 288 μm × 288 μm size were used for quantification. **E.** Quantification of vessel parameters: total length, number of junctions and branches in peritumoral area (TZ II) versus sham operated brain slices. Images with 570 μm × 570 μm size were used for quantification (*n* = 6). **F.** Distribution of vessels diameter (μm) in TZ II and sham (*n* = 6). **G.** Images represent tan assembly of pathological tumor vessels found in the VOGIM. Scale bars is given as 40 μm. Statistical analysis was performed with Student's *t*-test (*P < 0.05, error bars represent mean ± s.e.m.).

Hence, we investigated the tumor microenvironment in glioma-infiltrated brain tissue. For this we implanted red fluorescent protein-expressing tumor cells in brain slices from CX3CR1-GFP mice (Figure 4A). CX3CR1-GFP mice express GFP under the macrophage/microglia-specific promoter CX3CR1 thus enabling time lapse microglia movement tracking *in vivo* as well as visualization of perivascular microglia and their relation to newly formed vessels. After fixing and immunostaining in free-floating, VOGIMs were mounted onto microscopic slides. Brain tumors form a specific tumor microenvironment which consists of different heterogeneous areas with at least three different tumor zones termed TZ I-III [26]. Within this concept TZ I represents the tumor bulk whereas TZ II displays the peritumoral or perifocal area characterized by microglial activation and neuronal cell damage surrounding the tumor bulk. TZ III is revealed as the area following the perifocal area containing only few single tumor cells regarded as cancer stem cells or cancer initiating cells (Figure 4B, Supplementry Figure 2). Therefore, we examined blood vessels in the peritumoral area (TZ II) in comparison to similar areas which were sham treated (i.e. implanted with same volume of cell free medium) (Figure 4A). Interestingly, when we analyzed the neuronal distribution we found a clear pattern of tumor zones with a rim of cell damage surrounding the tumor bulk (Figure 4B, Supplementry Figure 2).

Next, we quantified microglia in peritumoral area versus sham treated controls. These experiments demonstrated that increased number of microglia is present in peritumoral area with contact or close proximity to vessels compared to controls (Figure 4C, 4D). Interestingly, when monitoring microglial cells in tumor-bearing brains we found increased numbers of microglia in peritumoral areas area (Figure 4C).

Further we facilitated the VOGIM to assess tumor-induced vascularization in the peritumoral area (TZ II) (Figure 4C, 4E). Staining brain slices with laminin illustrated a high density of vessels in peritumoral area (Figure 4C, 4E).

We also quantified total length, vascular junctions and branches in the peritumoral area. All three vascular parameters were increased in the peritumoral area (Figure 4E). Analysis of blood vessels diameter and distribution showed significant differences between peritumoral and control areas. In the peritumoral area we found a mean diameter range between 4 to 6 μm whereas in sham controls diameter range were between 2 to 4 μm (Figure 4F).

Analysis of tumor angiogenesis revealed different types of vessel pathologies. Tumor vessels appeared abnormal with tortuous, saccular and disorganized structures and were often found in proximity of a network of micro-vessels (Figure 4G). Noteworthy, these pathological tumor-induced vessels found in the VOGIM resembled the spectrum of pathological vessels also found in *in vivo* studies [27].

**Figure 5:**
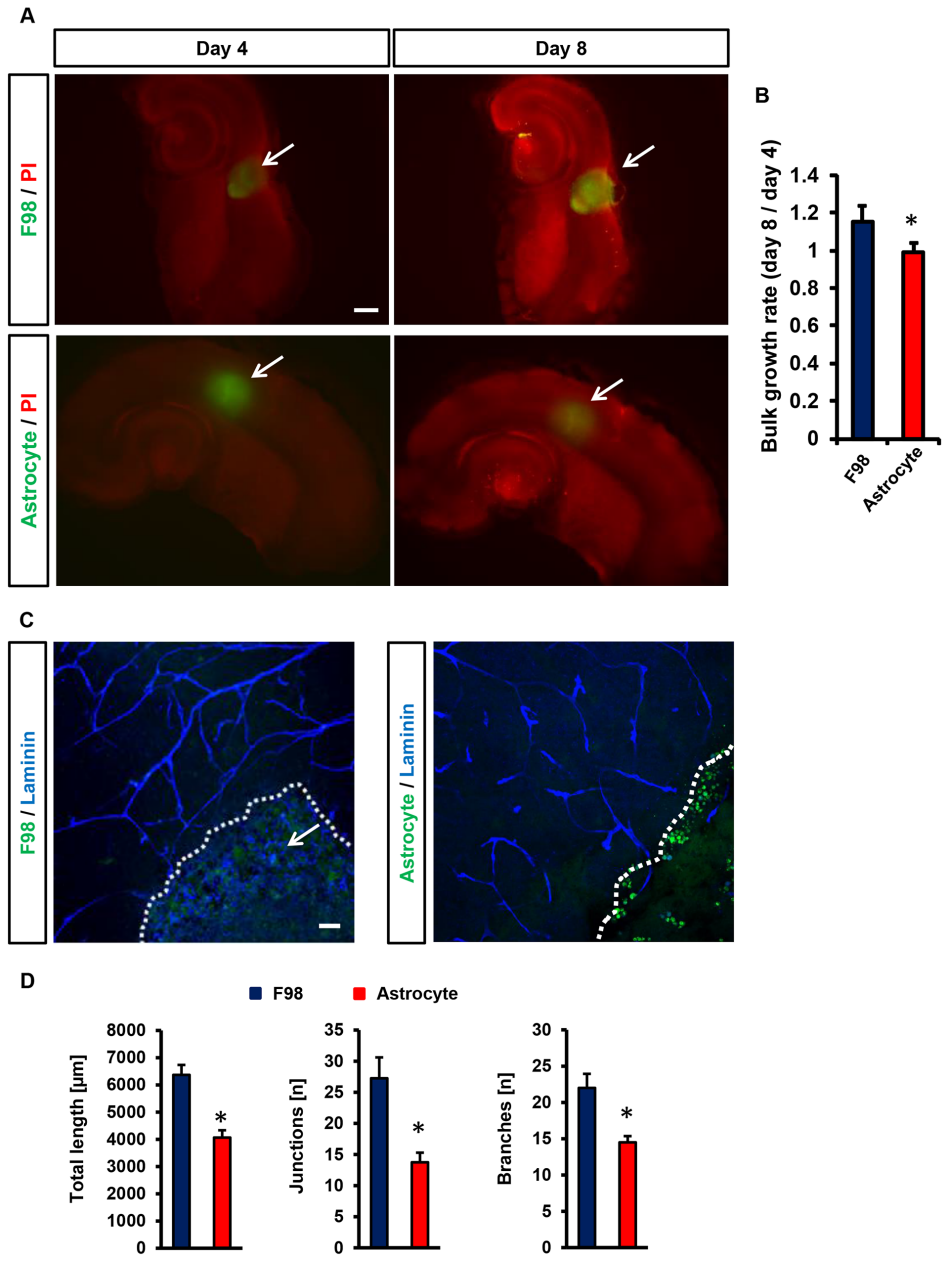
Vascular and cellular response after non-transformed cell implantation in VOGIM **A.** PI staining (red) and GFP positive area to monitor cell death and tumor growth respectively in brain slices (top) in comparison to astrocytes cell implantation (bottom). Given are cell growth at day 4 and 8. Arrows indicate tumor bulk. Scale bar represents 500 μm. **B.** Quantification of bulk cell growth area of gliomas (F98) and primary astrocytes in brain slices. **C.** Vascularization in peritumoral area (TZ II). Vessels are stained for Laminin (blue). Glioma cells (F98, left) or astrocytes (right) are displayed in green and cell growth is indicated by the white dotted line. Arrows indicate intra-tumoral vessels in glioma-implanted brains. Scale bar represents 50 μm. **D.** Quantification of vessel length, junctions and branches in glioma implanted slices (green) and astrocytes-implanted brain slices (green). Images with 288 μm × 288 μm size were used for quantification. Statistical analysis was performed with Student's *t*-test (*P < 0.05, error bars represent mean ± s.e.m, *n* = 6).

To validate the VOGIM assay we continued with a comparison of the impact of malignant cells and non-transformed cells in the brain environment. For this we implanted gliomas into brain slices and compared the results with that of implanted primary astrocytes (Figure 5). In the case of glioma cells tumor volume and peritumoral cell death increased over time and reached highest levels at day 8 in culture (Figure 5A). In contrast, cell death analysis of astrocytes-implanted brain slices revealed almost no alteration (Figure 5A). Furthermore, tumor size measurements displayed a significant increase in the case of gliomas (Figure 5B). In contrast, implanted astrocytes showed almost no bulk growth over time (Figure 5B).

Hence, we investigated the impact of implanted astrocytes in brains in comparison to gliomas (Figure 5C, 5D). Noteworthy, primary astrocytes did neither affect vessel length, junctions nor branches which remained at the level of control brain slices (Figure 5C, 5D). Thus, despite the implantation procedure and volume load non-transformed cells did neither alter cell damage nor the vasculature in the VOGIM (Figure 5C, 5D).

**Figure 6:**
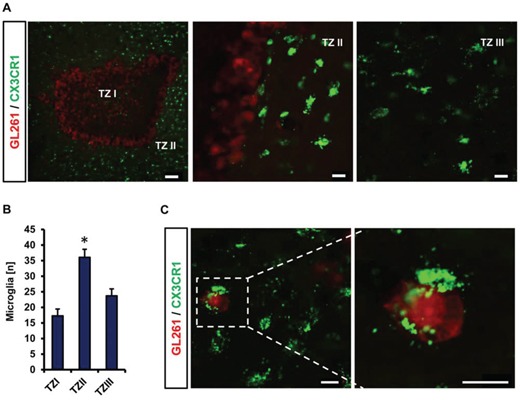
Microglia distribution and interaction in different tumor zones **A.** Distribution of microglial cells (green) located in tumor zone I, II and III. RFP stable expressing GL261 (red) were implanted into CX3CR1-GFP positive brain slices. TZ I represents the tumor bulk, TZ II indicates the peritumoral zone and TZ III is the area distant to TZ I. Scale bar represents 100 μm (for left), 20 μm for middle and right images. **B.** Quantification of the number of microglial cells in different tumor zones. Images with 710 μm × 710 μm size were utilized for quantification. Statistical analysis was performed with Student's *t*-test (*P < 0.05, error bars represent mean ± s.e.m, *n* = 4). **C.** Representative image displaying interaction of microglia with glioma cells. Scale bar represents 20 μm.

We continued to monitor microglial cells in the VOGIM assay. Therefore, we implanted red fluorescent protein-expressing glioma cells into brain slices from CX3CR1-GFP transgenic mice to track the movement and interaction of microglia with tumor cells during the culture phase (Figure 6). We monitored microglia cells in different tumor zones from TZ I to TZ III (Figure 6A). Tracking microglia displayed the highest number of microglia in the peritumoral area (TZ II) (Figure 6A, middle picture) while conspicuous alteration in number of microglia was detected in TZ I (Figure 6A, left picture). In comparison to TZ II, less microglia cells accumulated in TZ I (Figure 6B). Likewise, the number of microglia cells in TZ III was lower compared to TZ II (Figure 6B). In fact, microglial density in TZ III revealed comparable numbers to that found control brain slices (without tumor implantation) (data not shown). Furthermore, we monitored microglia cells engulfing glioma cells in peritumoral area (Figure 6C). These data indicate that the VOGIM technique is also suitable to study tumor-immune interactions with resident immune-competent cells *ex vivo*.

### Drug sensitivity and adverse reaction analysis in the VOGIM assay

**Figure 7:**
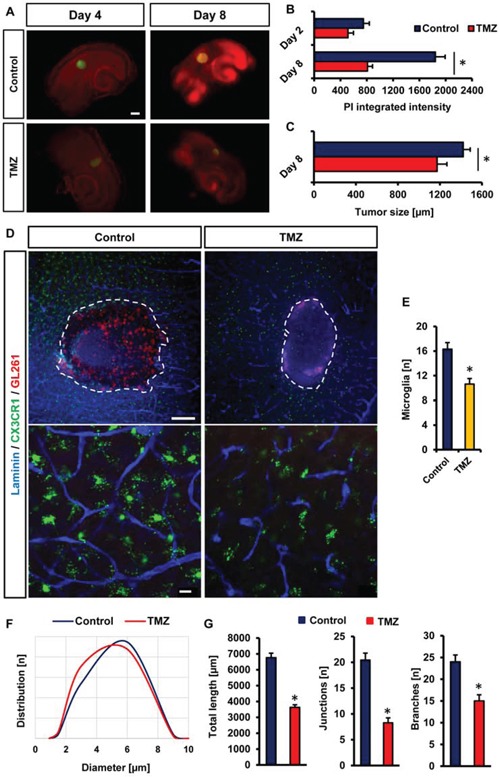
Analysis the drug sensitivity of VOGIM method **A.** PI staining (red) and GFP positive area (green) monitors cell death and tumor growth respectively in brain slices treated with temozolamide (TMZ, 100 μM). Untreated brain slices served as control samples and analysis was performed on day 4 and 8. Scale bar displays 500 μm. **B.** Quantification of cell death after temozolamide (TMZ) treatment in comparison to untreated control samples on day 4 and 8. **C.** Quantification of tumor growth after temozolamide (TMZ) treatment in comparison to untreated controls at day 8. **D.** Peritumoral (TZ II) vascularization of TMZ treated brain slices (CX3CR1-GFP positive) and untreated controls. Laminin staining (blue) was utilized for vessel visualization. Microglial cells are displayed in green. Scale bar in top images represents 200 μm, scale bar in bottom images shows 20 μm. **E.** Quantification of the numbers of perivascular microglial cells in peritumoral area (TZ II) of temozolomide (TMZ) treated and untreated tumor-implanted brain slices. Images with 288 μm × 288 μm size were used for quantification. **F.** Distribution of vessel diameters (μm) in peritumoral areas after temozolomide (TMZ) treatment and in untreated controls. **G.** Quantification of vessel length, junctions and branches in glioma implanted untreated slices and temozolomide treated brain slices. Statistical analysis was performed with Student's *t*-test (*P < 0.05, error bars represent mean ± s.e.m, *n* = 6).

Various protocols have been developed to evaluate the applicability of drugs or small molecule inhibitors on glioma cells and their microenvironment. In particular, vascularization and the tumor-immune interaction have become center of interest in recent years. Therefore, we went on to test whether the VOGIM is capable to display changes in tumor cell viability and microenvironmental alterations under specific drug treatment. One of the anti-cancer drugs which have been widely used and established as standard for the treatment of glioblastoma is Temozolomide (TMZ, Temodal/Temcad^®^). TMZ is a chemotherapeutical drug belonging to the alkylating agents transferring a methyl group to purine bases of DNA (O6-guanine; N7-guanine and N3-adenine). In contrast to many other chemotherapy drugs, TMZ can reach the brain via a systemic route and is nowadays standard in glioblastoma therapy. Therefore, we used this drug and examined the effects on peritumoral vascularization. One day after tumor implantation, we started with TMZ treatment in the VOGIM with 100 μM TMZ every second day. The experiment was determined on day 8 and slices were further processed for analysis. Treatment of tumor-implanted brain slices with TMZ led to reduced cell death compared to untreated controls (Figure 7A, 7B). Moreover, tumor size measurements revealed reduced tumor growth under TMZ treatment (Figure 7C). We further examined peritumoral vascularization and microglial distribution in TZ II under TMZ treatment (Figure 7D). Interestingly, the number of microglial cells decreased in TZ II after TMZ application (Figure 7E). Furthermore, determination of vessel diameters of peritumoral vessels showed that TMZ treatment slightly led to smaller vessels (Figure 7F). Moreover, TMZ treatment reduced significantly overall vascularization parameters towards normalization (Figure 7G).

**Figure 8:**
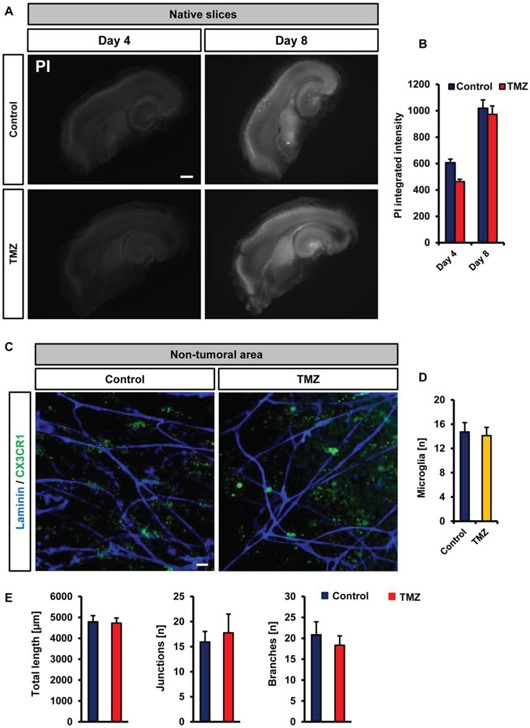
Vascular and cellular response after temozolomide treatment in VOGIM **A.** Cell death monitoring (PI staining, white signal) in native slices under untreated control conditions (top) and after temozolomide (TMZ, 100 μM) treatment (bottom). Given is cell death at day 4 and day 8. Scale bar represents 500 μm. **B.** Quantification of cell death in brain slices after temozolomide (TMZ, 100 μM) treatment. Untreated controls are displayed in blue, TMZ treated group is given in red. **C.** Vascularization and microglial distribution of brain areas after temozolomide (TMZ) treatment. Vessels are stained for Laminin (blue) and microglial cells are displayed in green. Scale bar represents 20 μm. **D.** Quantification of the numbers of perivascular microglial cells after temozolomide (TMZ) treatment and in untreated (control) brain slices. **E.** Quantification of vessel length, junctions and branches in brain slices treated with temozolomide (TMZ) or untreated (control). Images with 288 μm × 288 μm size were used for quantification. Statistical analysis was performed with Student's *t*-test (*P < 0.05, error bars represent mean ± s.e.m, *n* = 6).

We went on and investigated the TMZ effects on native brain tissue. We found that TMZ did not increase neuronal damage compared to untreated controls (Figure 8A, 8B). We continued to monitor also the effects of TMZ on microglial cells and the vasculature. Although TMZ was highly efficient on alleviating glioma-induced alteration, TMZ did neither affect microglial distribution, morphology nor vasculature in normal brain tissue (Figure 8C–8E).

Taken together, our data propose the VOGIM technique is appropriate to investigate the impact of chemotherapeutics on tumor growth, cell survival, angiogenesis and microglial cells.

## DISCUSSION

Here, we describe the establishment of the Vascular Organotypic Glioma Impact Model (VOGIM) as a robust and reliable tool to investigate physiological and pathological angiogenesis. In principle, any brain tissue from wild type or transgenic mice or rodents can be facilitated for the organotypic brain slice assay. We provide evidence that the *ex vivo* cerebral vasculature and the intact cell structure resemble closely the *in vivo* environment. The VOGIM is based on the trans-well interface method [28–32]. The VOGIM requires solely standard equipment and is a straight and easy-to-perform method. Different genetic strains and transgenes can be used for donor tissues with respective littermate controls. For controls genetically identical slices with different treatment regiments can be compared. Also, the VOGIM can be prepared virtually from any region of the brain [22] or even from peripheral organs like liver, kidneys and many others [23]. The VOGIM allows analysis of neurons, glial cells and the tumor-microenvironment in an *in vivo*-like, complex system with preserved tissue architecture similar to that seen in the living. Tumor growth, optical and biochemical assessment of the microenvironment can be conducted by implanting reporter gene expressing glioma cells (i.e. fluorescent protein expression such as GFP, RFP, CFP and others). Tumor cells adhere and invade into the brain slice, form a tumor bulk and attract and induce vessels by secreting angiogenic factors. Due to the organotypic and anatomical preservation tumor zone II (TZ II according to the brain tumor zone model [26]) is highly affected by tumor-induced angiogenesis showing an aberrant vessel architecture and a high-dense micro-vascular network of newly formed vessels [33–35]. Induced angiogenesis can be analyzed by quantitative means such as vessel density in defined areas, vessel diameters, number of branches ranging from a parent vessel and vessel length. Moreover, monitoring of defined cells in brain slices can be achieved by acquiring transgenic animals expressing live-cell reporter genes in a cell-type specific manner such as CX3CR1 (for microglia), GFAP (for astrocyte-specificity) or tie1 (for endothelial cells) and by ectopic gene expression through ectopic transfection or viral infection [24]. Furthermore, the impact of tumors on neurons and bystanders is assessable in the VOGIM [11, 25, 36].

In physiological processes such as during development or reorganizational processes angiogenesis is active in a transient mode as a self-limiting process [37, 38]. Conversely, in pathologies such as stroke, inflammation, epilepsy, Alzheimer's disease and glioblastomas vessels are dynamic and as a result the vascular architecture is altered and shows aberrant structures and pathological vessels are formed [39–41]. In particular, primary brain tumors are highly angiogenic active with constant secretion of growth-promoting and pro-angiogenic factors. Interestingly, pathological tumor-induced vessels found in the VOGIM resembled those pathological vessels found *in vivo* [27]. These similarities with human conditions are noteworthy since perfusion, intravasal pressure dynamics and functional blood flow are absent in the VOGIM. There are advantages in evaluating the angiogenic process and the vascular morphology in organotypic 3D-cultures compared to 2D cell culture. Assays like the tube formation assay [20, 21] which facilitates endothelial cells (HUVECs or brain endothelial cells) are to a certain extent redundant since these cell models are firstly dissociated from their natural environment and secondly deprived from connectivity-dependent signals and their overall organotypic environment. In contrast, the VOGIM allows the analysis of neurons, microglial cells and the tumor-microenvironment in an *in vivo*-like, complex system with preserved tissue architecture, connectivity and signals similar to that seen *in vivo*.

Tumor growth and micro-environmental studies can be performed by implanting reporter gene expressing malignant glioma cells (i.e. various fluorescent protein expressions such as GFP, RFP, YFP, CFP and others). Tumor cells adhere and invade into the brain slice, form a tumor bulk and attract vessels by secreting angiogenic factors.

Another advantage of this *ex vivo* model is that the VOGIM enables pharmacological testing of virtually any drug compound in real time mode. These drugs could target the tumor angiogenesis as well as the tumor growth. The VOGIM allows the simultaneous analysis of drug impact on vessels and tumor microenvironment [25, 42–44]. In particular questions of unintended side effects and bystander effects can be investigated simultaneously by this method [43]. This extents the investigations of novel drugs beyond expected target analysis.

Angiogenesis can be assessed quantitatively in defined areas in various tumor zones and peritumoral regions. Here, we compared two established and widely used methods for the analysis of the vasculature. Both the grid-overlay method as well as software-based applications assessed tumor vessels in a quantitatively reliable manner and reflected the angiogenic complexity in different tumor zones. As revealed by the VOGIM the tumor bulk and its secreted factors have an enormous influence on the viability of brain tissue and neuronal function [25, 36]. Secreted factors attract vessels and the vessels grow around the tumor bulk in an accumulating fashion. Despite the fact, that no bloodstream is flowing and perfusion is absent in the cell culture, the vital brain tissue and its angiogenic reactions and adaptations include all required components in its organotypic organization. Also, endothelial cells and pericytes remain viable (data not shown) for the whole culture time in the VOGIM.

The VOGIM procedure, which we described here, can be used to generate slice cultures from virtually any part of the brain. It is crucial that the normal brain morphology and connectivity is retained upon slicing. Also, the VOGIM can be facilitated to monitor glioma invasion and migration [45, 46]. The VOGIM in particular strives to combine the versatility of tumor proliferation, microenvironment and angiogenesis assays with complexity of the organ-specific situation. Furthermore, the VOGIM can well be considered as a new technique in the angiogenesis field implementing the 3Rs for the sake of reducing animal experiments. Altogether, we suggest the *ex vivo* brain slice model, the VOGIM, as a reliable alternative to many *in vitro* assays and *in vivo* trials.

## MATERIALS AND METHODS

### Cell culture

Rodent glioma cell line F98 and mice glioma cell line GL261 were obtained from ATCC/LGC-2397 (Germany). All cell lines were cultured under standard humidified conditions (37°C, 5% CO_2_) with Dulbecco's Modified Eagle Medium (DMEM; Biochrom, Berlin, Germany) supplemented with 10% fetal bovine serum (Biochrom, Berlin, Germany), 1% Penicillin/Streptomycin (Biochrom, Berlin, Germany) and 1% Glutamax (Gibco/Invitrogen, California, USA). Cells were passaged at approx. 80% confluence. For dissociation, glioma cells were washed with PBS for 3 min and then treated with trypsin 0.05% at 37°C for 3 min and collected by gentle pipetting.

### Vascular organotypic brain slice cultures (VOGIM) and cell death monitoring

Four-day-old Wistar rats (Charles River, Boston, MA, USA) or CX3CR1-GFP transgenic mice were decapitated. Brains were cautiously removed and maintained under ice-cold conditions. Frontal lobes and cerebellum were dissected off. The remaining parts of the brain were glued to specimen discs. A vibratome (Leica VT 1000S, Bensheim, Germany) was used to cut horizontally the brain into 350 μm thick slices. Thereafter, brain slices were collected and trimmed. The midbrains were cut off and discarded and the hippocampus slices were transferred onto culture plate insert membrane dishes (Greiner BioOne, Frickenhausen, Germany; pore size 0.4 μm) and subsequently transferred into six-well culture dishes (GreinerBioOne, Frickenhausen, Germany) (Figure 1A). Brain slices were cultured at humidified conditions (35°C, 5% CO_2_) with 1.4 ml culture medium per well included MEM–HBSS (Gibco/Invitrogen, California, USA), 2:1, 25% normal horse serum (Biochrom, Berlin, Germany), 2% L-glutamine (Gibco/Invitrogen, California, USA), 2.64 mg/ml glucose (Merk, Germany), 100 U/ml penicillin (Sigma-Aldrich), 0.1 mg/ml streptomycin Sigma-Aldrich), 10 μg/ml insulin–transferrin–sodium selenite supplement (Invitrogen, California, USA) and 0.8 μg/ml vitamin C (Merk, Germany). On the first day and before tumor implantation in culture, a cell death staining was performed with propidium iodide (Sigma-Aldrich) (0.1 mg/ml) and the quality of the acquired brain slices determined (Figure 1B). After PI-staining, the slices were washed once with 1 M phosphate buffered saline and a complete medium exchange was performed thereafter. After cell death measurement, tumor cells, astrocytes or buffer volume were implanted into the slices (100,000 cells/0.1 μl medium per slice) with a micro-syringe (Figure 1B). Astrocytes were prelabelled with DiI (Molecular Probes, USA) for at least 8 hours prior implantation. Crucial for the tumor implantation procedure is a sensible handling of the brain slices and the tumor cells. The brain slice must not be damaged during the tumor implantation. The procedure of cell death staining, washing and complete medium exchange was performed consecutively every other day over a course of 8 days. A time-lapse cell death analysis can be performed after the culture period.

### Immunofluorescence staining

After the slices were cultured for 8 days, the slices are fixated with para-formaldehyde (Merk, Germany) for 45 to 60 minutes, followed by permeabilization for 30 minutes with 0.5% Triton-X (Sigma-Aldrich) in PBS. Primary antibodies laminin (Sigma-Aldrich) were diluted in a concentration of 1:1000 in 3% horse serum (HS) or fetal calf serum (FCS) and 0,2% Triton X-100 as the blocking solution and subsequently incubated for overnight at 4°C. Alexa Fluor 405, 448 and 568 (Invitrogen/Life Technologies) were used as secondary antibodies with 1:1000 concentration. Slices were then washed twice in PBS, stained with Hoechst (Invitrogen/Life Technologies) and again washed in PBS. Then, stained tissue slices were flat-mounted with ImmuMount (Thermo Scientific).

### Vascular quantification methods

The overlaying grid method was used to analyze the density of the vessels. After culturing, fixation and immunohistochemical vessel-staining, the peritumoral zone and control zones in each brain slice are defined. Images are acquired with a fluorescent or confocal microscope. Since the peritumoral zone is a narrow zone directly around the tumor bulk, a higher magnification of x40 or higher is recommended. The acquired images are analyzed with the grid method. With Image J or Adobe Photoshop a stereological grid consisting of 12 squares with the side lengths of 80 μm is superimposed onto the experiment images of the brain slices stained for laminin. The density of blood vessels in the tissue is calculated by counting the intersections of blood vessels and virtual grid. The number of intersections of vessels crossing the outlines of a defined grid was counted as well as the number of intersections of vessels and inner grid lines. If two different vessels seem to cross the grid in one intersection point, the intersections of the both vessels are counted. The total number of vessels crossing the grid correlates with the level of vessel density and angiogenesis. All intersections of vessels and grid area are counted.

The *Angiogenesis Analyzer* is an Image J based tool to quantify experimental images and allows analysis of cellular networks (http://image.bio.methods.free.fr/ImageJ/?Angiogenesis-Analyzer-for-ImageJ.html, documents downloadable at: http://image.bio.methods.free.fr/ImageJ/?Angiogenesis-Analyzer-for-ImageJ.html). After fixation and vessel staining, control zones and peritumoral zone are defined. Images are acquired with the help of a fluorescent or confocal microscope. As far as the magnification is concerned, the analysis should be performed at higher magnification, since the peritumoral zone is a very narrow and close to the tumor bulk area. Images acquired in the closest distance at these magnifications, exactly show the vessels of the peritumoral zone. After collecting, the experimental images are handled with the *Angiogenesis Analyzer*. Using binary thinning procedures, the image is skeletonized. Through further processing, characteristic information of the vessel network is extracted and branching points and meshes are identified. We obtained the following parameters: total length, number of junctions and number of branches. Locating branching points or nodes in the skeleton is performed by locating pixels with more than 2 pixel-neighbors. The meshes or the number of meshes correlates with the number of branches and the interconnection of the vessels. The total length is performed by splitting off all lines between branching points or between branching points and image border, then the length of each line are summed up. Mainly, this software application is used in anti-angiogenic drug trials in combination with human umbilical vein endothelial cells (HUVECs). We adapted the application and its macros for analyzing the vessel network in the VOGIM.

### Temozolomide treatment

One day after tumor implantation, temozolomide (Sigma-Aldrich) was added to the culture medium at a final concentration of 100 μM and was renewed every second day with changing culture medium within 6 days. Temozolomide was dissolved in dimethyl-sulfoxide (DMSO) which we used the same amount of DMSO as vehicle control.

### Statistical analysis

Quantitative data from experiments were obtained as stated in the figure legends. Analysis was performed using unpaired Student's *t* test (MS Excel). Data from all experiments were obtained from at least three independent experiments. The level of significance was set at * indicates *p* < 0.05. Error bars represent ± s.e.m. if not otherwise stated.

## SUPPLEMENTARY FIGURES



## Abbreviations

3Rs: Three Rs (Replacement, Reduction, Refinement)
GFP: green fluorescen protein
HUVEC: human umbilical vein endothelial cells
MG: microglia
PI: propidium iodide
TMZ: temozolamide (trade names are Temcad/Temodal)
TZ: tumor zone
VOGiM: Vascular organotypic glioma impact and invasion model.
