# Supplementary material for: A versatile ex vivo technique for assaying tumor angiogenesis and microglia in the brain
Source: Oncotarget. 2015 Dec 11;7(2):1838–53. doi: 10.18632/oncotarget.6550 (PMC4811501; doi:10.18632/oncotarget.6550)
Supplement: Supplementary file 1 [file oncotarget-07-1838-s001.pdf]

SUPPLEMENTARY FIGURES

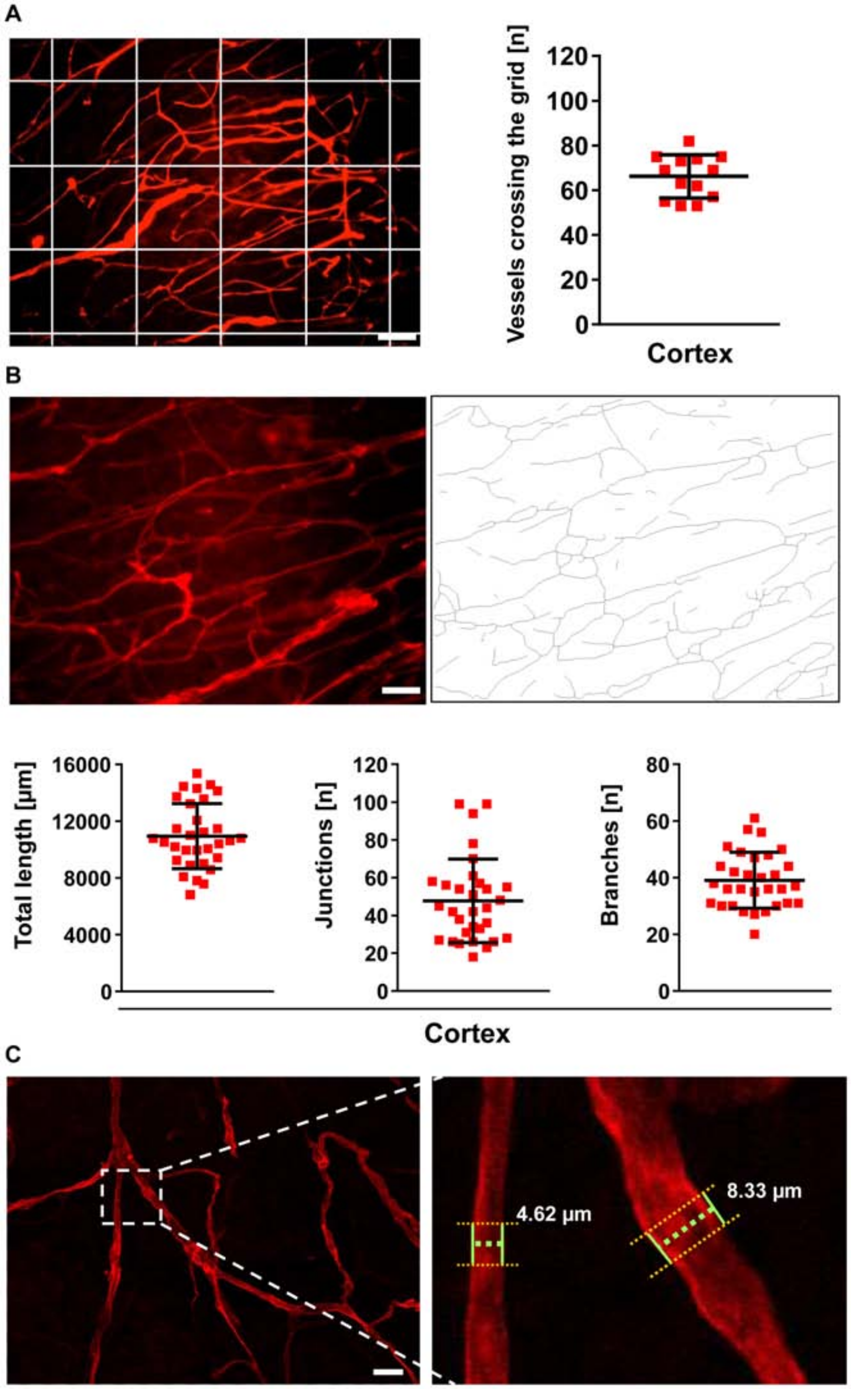

Supplementary Figure S1: Quantitative assessment of various established angiogenesis analysis tools. Divergent approaches for the analysis of vasculature are given. (**A.** right panel) The grid method for vessels density measurement is shown. (**A left panel**) Quantification of vessels with the grid method representing number of vessels crossing the grid. Scale bar represent 20 µm (*n* = 9) **B.** Quantification different vascular parameters with the Image J software and Angiogenesis tools plugin. We considered three parameters relevant in tumor angiogenesis and physiology: Total vessels length, number of junctions and branches. Scale bars represents 20 µm. **C.** Vessels diameter measurement in length of vessels with Image J and ZEN software (Carl Zeiss). Scale bar represents 20 µm. Error bars represent SD value.

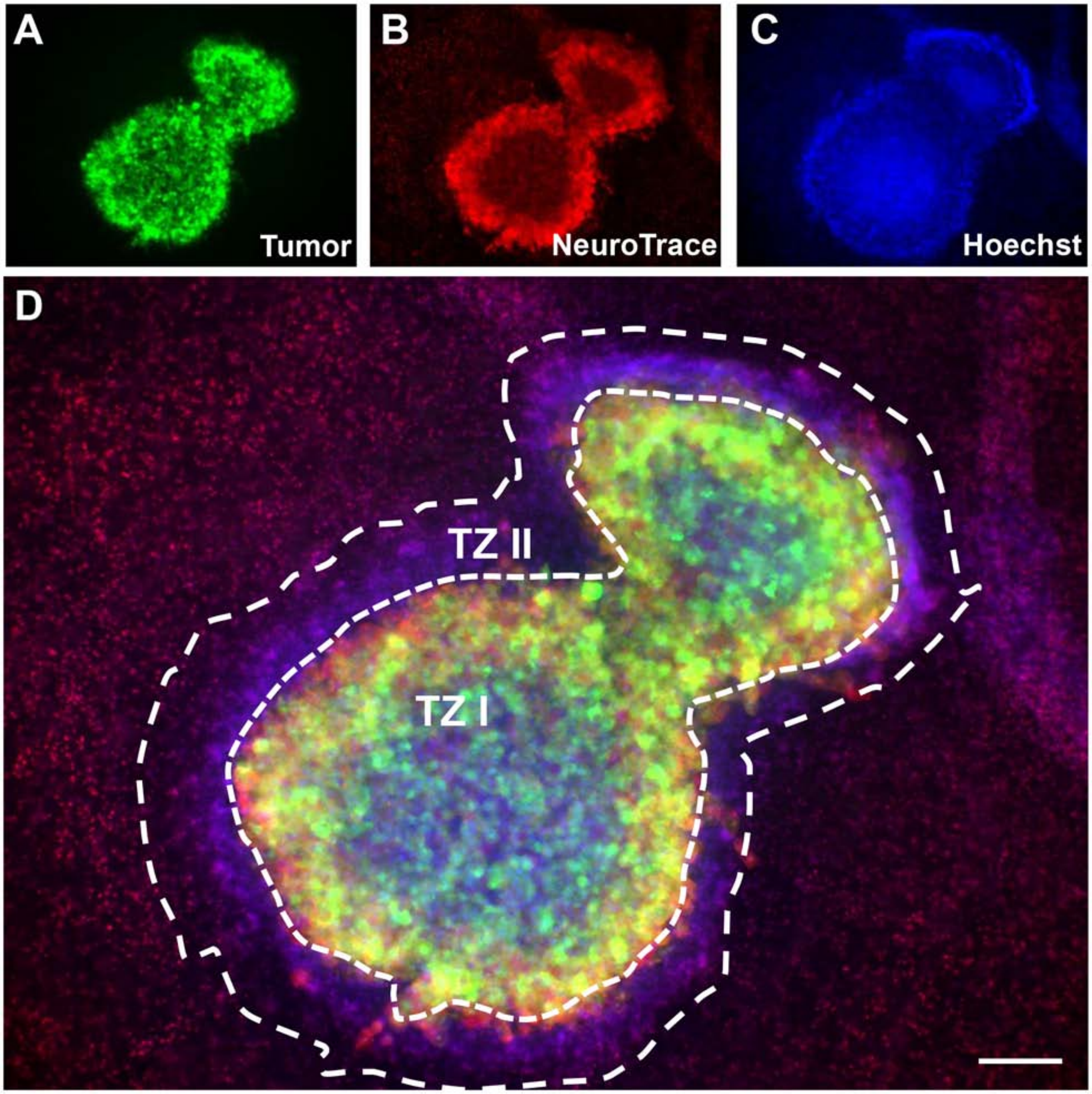

Supplementary Figure S2: Analysis of cell death and tumor zones in the VOGIM. A. GFP signal (green) of a tumor-implanted brain slice. After tumor implantation, glioma cells form a bulk and secrete factors shaping their specific tumor microenvironment. **B.** A particular peritumoral zone (TZ II) appears challenged with disturbed neuronal layering. The peritumoral zone is characterized by a rim-like structure around the tumor bulk. Representative image of the same slice stained for NeuroTrace (red). NeuroTrace stains specifically neurons or neuronal-associated endoplasmatic reticulum. Note, that neurons within the TZ II appear disrupted and show soften and blurry staining characteristics and lose their typical punctuated appearance. **C.** Total cell nuclei staining is shown (blue, stained with Hoechst 33258). **D.** Representative merged image of A-C. Total cell nuclei staining is shown (blue, stained with Hoechst 33258), tumor (green) and neurons (red) are given. The merged picture shows the typical breaking-up of the neuronal cell structure in the peritumoral TZ II zone. Scale bar represents 100 µm.
